# Targeting β-catenin: PROTACs and precision degraders for Wnt-driven cancers

**DOI:** 10.3389/fonc.2026.1777843

**Published:** 2026-02-02

**Authors:** Jonathan Trapani, Kailey P. Caroland, Yashi Ahmed, David J. Robbins, Vivian L. Weiss, Ethan Lee

**Affiliations:** 1 Department of Cell and Developmental Biology, Vanderbilt University, Nashville, TN, United States; 2 Department of Pathology, Microbiology, and Immunology, Vanderbilt University Medical Center, Nashville, TN, United States; 3 Department of Molecular and Systems Biology, Geisel School of Medicine, Dartmouth College, Hanover, NH, United States; 4 Department of Oncology, Lombardi Comprehensive Cancer Center, Georgetown University, Washington, DC, United States; 5 Vanderbilt Ingram Cancer Center, Vanderbilt University School of Medicine, Nashville, TN, United States

**Keywords:** beta-catenin, drug discovery, PROTAC (proteolysis targeting chimera), toxicity, Wnt

## Abstract

The Wnt signaling pathway, a highly conserved molecular cascade, orchestrates critical biological processes including embryonic development, cell differentiation, and proliferation across diverse organisms. Despite the pivotal role that Wnt signaling plays in many diseases, most notably cancer, there are still no FDA-approved, efficacious drugs available that inhibit this pathway. Most Wnt inhibitors target upstream components (e.g., Wnt ligand production and receptors) rather than the most commonly mutated downstream proteins in the pathway. Consequently, there is considerable interest in developing drugs that target the downstream effector, β-catenin. This review examines the challenges in targeting β-catenin, current approaches, and insights into overcoming on-target toxicity associated with cadherin-bound β-catenin.

## Introduction

The Wnt signaling pathway plays an important role in human development, the maintenance of the adult organ stem cell niche, and is dysregulated in human disease (1–13). Aberrant activation of the canonical (Wnt/β-catenin) signaling pathway drives the progression of many diseases, particularly cancer (14–23), yet no effective therapeutics targeting this pathway have been developed. While there are a few Wnt inhibitors in clinical trials, they primarily target proteins that function upstream of the commonly mutated components, limiting their efficacy (24–29). Consequently, targeting the transcriptional co-activator β-catenin, the primary downstream effector, holds significant clinical promise. Yet, obstacles to developing therapies that inhibit β-catenin include the paucity of targetable grooves in β-catenin for small-molecule binding and β-catenin’s crucial physiologic role in adherens junctions. Disruption of β-catenin’s role at the membrane in adherens junction formation would likely result in unacceptable dose-limiting on-target toxicity (30–33). Emerging classes of protein degraders, including proteolysis-targeting chimeras (PROTACs), have the potential to target the previously considered “undruggable” cytoplasmic β-catenin and to evade degradation of membrane-bound, cadherin-associated β-catenin. While previous reviews covering canonical (Wnt/β-catenin) pathway inhibitors have been published (34–40), there have been few updates on new technical advances in targeting β-catenin. Given the rapid evolution of novel drug classes and the urgent need to develop β-catenin-targeting therapeutics, this review provides a timely update.

## The Wnt signaling pathway

### Mechanism

Wnt signaling directs development in metazoans, including freshwater hydra, the sea sponge Oscarella, flies, frogs, and mammals, including humans (41–43). One of the first Wnt components discovered was in Drosophila in 1973 (3, 4). Named *wingless* (*wg*), mutations in this gene prevented wing specification. The mammalian oncogene *Int-1* was discovered in 1982 and found to be the ortholog of the Drosophila *wingless* gene in 1987 (44, 45). The names of the Wingless and Int-1 proteins were combined to form the portmanteau “Wnt”. Subsequently, the Adenomatous Polyposis Coli (APC) tumor suppressor protein, which is frequently mutated in colorectal cancers, was found to regulate Wnt signaling (46–49). These discoveries highlighted the multifaceted role of the Wnt pathway in animal development and human disease, sparking significant research interest.

The Wnt signaling system includes the canonical (β-catenin-driven (50–55)) and non-canonical (calcium (56, 57), planar cell polarity (58–61), and Wnt/STOP (62, 63)) pathways. Wnt/β-catenin signaling is activated by Wnt ligands binding to the Frizzled family of receptors and LRP5/6 co-receptors, while LRP5/6 are considered dispensable for non-canonical (non-Wnt/β-catenin) signaling. There are 19 Wnt ligands and 10 Frizzled receptors (64, 65), with the specific ligand-receptor pairs determining the activated pathway (66). The Wnt/β-catenin (henceforth Wnt) pathway is most frequently linked to human disease when disrupted and centers around its key effector, β-catenin (18, 67–76).

**Figure 1:**
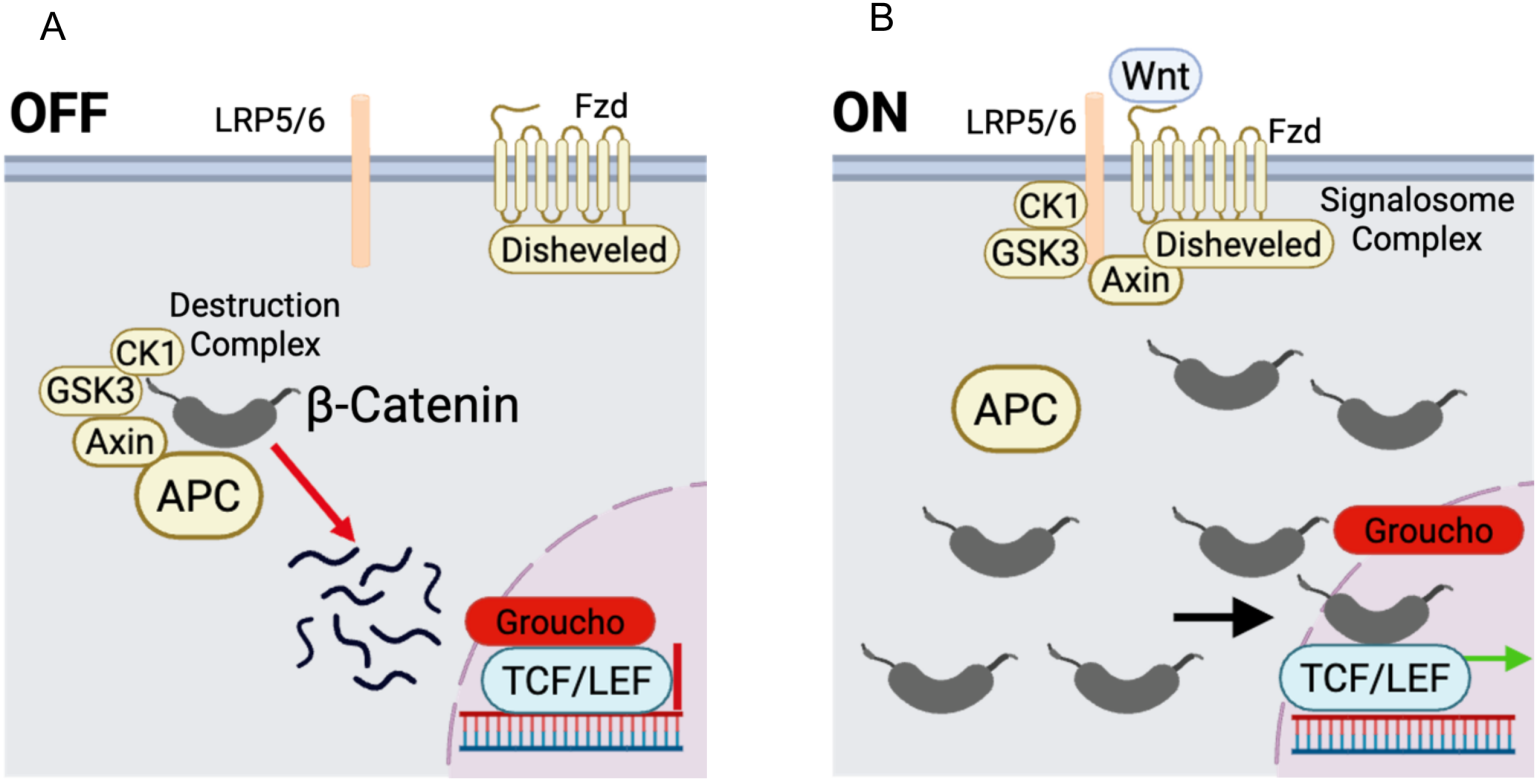
The Wnt signaling pathway. In the absence of Wnt, β-catenin is phosphorylated by the destruction complex, which is composed of CK1α, GSK3, Axin, and APC. This phosphorylation promotes the degradation of β-catenin by the ubiquitin/proteasome system **(A)**. In the presence of Wnt ligand, the destruction complex is disassembled, and β-catenin remains stable, enters the nucleus, and induces the Wnt transcriptional program **(B)**.

The Wnt pathway exists in two general states: “OFF” (no Wnt ligand present) and “ON” (Wnt ligand present). In the off state, cytoplasmic β-catenin is degraded by the destruction complex, a protein complex comprising APC, AXIN, GSK3, and CK1α (46, 77–79). APC and Axin act as scaffolding proteins that promote the recruitment of β-catenin and its phosphorylation by CK1α at S45, followed by processive phosphorylation by GSK3 at S33, S37, and T41 (80, 81). Phosphorylated β-catenin subsequently binds the F-box protein, β-TrCP, leading to β-catenin’s ubiquitination and proteasomal degradation (82, 83). In the absence of β-catenin, Groucho/TLE transcriptional factors inhibit the Wnt-driven transcriptional program (84) (**Figure 1A**). The binding of Wnt ligands to the Frizzled and LRP5/6 receptors induces the formation of an active, oligomerized receptor complex known as the signalosome, which relies on the cytoplasmic protein Dishevelled (85–89). Dishevelled multimerization at the plasma membrane provides a scaffold for clustering of Frizzled and LRP5/6 receptors (90–92). In the current model, the signalosome recruits the destruction complex components Axin and GSK3 (93–95). GSK3 and CK1 family members phosphorylate LRP5/6 at their five PPPSPxS sites, enhancing their interaction with destruction complex proteins (95–98). This interaction inhibits β-catenin phosphorylation, leading to decreased ubiquitination and proteasomal degradation and increased cytoplasmic β-catenin levels (83, 99). β-catenin subsequently translocates to the nucleus, displaces Groucho/TLE, binds to members of the TCF/LEF transcription factor family, and initiates a Wnt/β-catenin transcriptional program (53, 54, 84, 100, 101) (**Figure 1B**).

### Function and Diseases

The Wnt signaling pathway regulates key cellular processes essential for human physiology and potential therapeutic applications. It supports development and tissue maintenance by influencing lineage-specific differentiation, exemplified by activating *RUNX2* expression to promote osteoblast differentiation from mesenchymal progenitors (102, 103) and by promoting the differentiation of mature lung cells (104) and liver cells following injury (105). The pathway also facilitates cell migration during embryonic development (106, 107). Additionally, Wnt signaling modulates cellular metabolism by promoting glutamine catabolism to support protein synthesis and enhancing glucose uptake and glycolysis to meet high energy demands (108, 109). Finally, through the transcriptional regulation of *MYC* and *CCND1*, Wnt signaling drives cell proliferation and stem cell renewal (110, 111).

As Wnt signaling is crucial for normal development and functions across multiple physiological systems, it is not surprising that it is implicated in numerous disease states. Notable diseases associated with altered Wnt signaling components include bone density defects, skeletal abnormalities (e.g., Robinow syndrome), familial exudative vitreoretinopathy (a developmental disorder affecting retinal blood vessels), and developmental tooth defects (reviewed by Nusse and Clevers (112)). Wnt signaling is also implicated in neurological diseases, including Alzheimer’s disease, dementia, and autism spectrum disorder, as well as cardiac diseases, including heart malformations, coronary artery disease, and heart failure (113–116).

Despite the wide range of diseases associated with upregulated Wnt signaling, its most prominent role is in cancer. Colorectal cancer is the best-known Wnt-driven malignancy, with up to 92% of sporadic colorectal cancers harboring mutations in genes encoding components of the Wnt pathway (15). Approximately 80% of all sporadic colorectal cancers carry an *APC* mutation (14). In non-*APC* mutant tumors, many patients exhibit constitutively active *CTNNB1* mutations (15) or alterations in other Wnt pathway regulators, including R-spondin proteins, which stabilize Wnt receptors, as well as RNF43 and ZNRF3 proteins, which target Wnt receptors for degradation (117–120). Mutations that activate the Wnt pathway are not exclusive to colorectal cancer. Approximately 35% of hepatocellular carcinomas are driven by Axin inactivation or β-catenin activation (15). Similarly, hepatoblastoma, the most common pediatric liver cancer, nearly always carries activating *CTNNB1* mutations (16).

In addition to destruction complex components, Wnt ligands are also implicated in tumor progression across multiple cancers. For instance, aberrant expression of *WNT2B*, *WNT3A*, and *WNT5A* is associated with the development and progression of non-small cell lung cancer (17). In thyroid cancer, where Wnt pathway mutations are less common, tumorigenesis is partly driven by Wnt signaling via Wnt ligands (18, 19). Notably, in anaplastic thyroid cancer, the expression of *WNT1*, *WNT2*, *WNT5b*, *WNT6*, *WNT7a*, *WNT10a*, and *WNT10b* ligands are significantly elevated compared to benign samples (19). Additionally, in thyroid cancer, both tumor cells and cancer-associated fibroblasts contribute to Wnt ligand production and exhibit heightened Wnt signaling (18, 20). Given the diverse mechanisms and sources of upregulated Wnt signaling, the Wnt signaling pathway represents a compelling therapeutic target for a broad range of cancers.

### Wnt inhibitors

Numerous strategies have been explored to target the Wnt pathway. Mao et al. have recently published a summary of Wnt-inhibiting agents that have progressed to clinical evaluation (121). Completed trials for compounds such as Vantictumab (OMP-18R5), Ipafricept (OMP−54F28), and WNT974 (LGK974), which target Frizzled receptors (27, 122), Wnts (123–125), and Porcupine (126), respectively, showed a variety of adverse effects, including diarrhea, nausea, leukopenia, neutropenia, bone toxicity, dysgeusia, and bone fracture (121). Additionally, a clinical trial for SC-006, an antibody-drug conjugate targeting RNF43, was terminated due to serious adverse effects, including thrombocytopenia (127). Similarly, an antibody drug conjugate, septuximab, which targets Frizzled 7, has been developed that demonstrates promising results in preclinical models (128). Potential concerns about septuximab, however, include known toxicities associated with the drug conjugate (monomethyl auristatin E) and potential on-target toxicities associated with targeting Frizzled 7. Critically, because β-catenin lies downstream of the aforementioned targets, such agents are likely to be ineffective against activating mutations in the destruction complex or degradation-deficient mutant β-catenin. Efforts have also been made to demonstrate the potential of CK1α agonists, which inhibit Wnt signaling by promoting β-catenin degradation and also by targeting the Wnt nuclear factor, Pygopus (129). The prototypical CK1α agonist, Pyrvinium, an FDA-approved anthelmintic drug, has shown promise as an anti-cancer drug preclinically (19, 129–133). A clinical trial is currently underway to assess pyrvinium’s efficacy for pancreatic ductal adenocarcinoma (134). Regardless, because β-catenin is the main effector of the Wnt pathway, targeting β-catenin itself represents a compelling therapeutic strategy to target the pathway downstream of the common activating Wnt mutations found in human cancers.

There are currently active clinical trials for drugs targeting the interaction of β-catenin with its various transcription factor partners. For example, FOG-001, a peptide that competitively inhibits β-catenin’s interaction with TCF, is currently in a clinical trial for solid tumors with Wnt pathway-activating mutations (NCT05919264) (135). FOG-001’s ability to directly target the β-catenin-TCF interaction is a significant leap forward in targeting this pathway, resulting in FDA’s fast track designation in November 2025 for treating desmoid tumors. Other current trials are also testing drugs that inhibit β-catenin’s interactions with other transcription factors essential for Wnt activation, including BCL9, CBP, and/or TBL1 (121). Notably, ST316 antagonizes the interaction between β-catenin and BCL9 and is currently in Phase I/II clinical trials (ClincialTrials.gov ID: NCT05848739) (136). However, while clinical trials remain active for this class of β-catenin inhibitors, potentially dose-limiting adverse effects, including fatigue, diarrhea, and emesis, have already been reported (121).

### Directly targeting β-catenin

β-catenin is composed of an N-terminal regulatory domain, a central domain of 12 armadillo repeats, and a C-terminal domain (33). The N-terminal domain includes the phosphorylation sites S33, S37, T41, and S45, which serve as a docking site for the β-TRCP E3 ligase complex (81). β-catenin primarily interacts with other proteins via extended flat surfaces in its central armadillo repeat domain, which are more challenging to target than enzymatic grooves in other proteins (33, 137, 138). Targeting cadherin-bound β-catenin may pose a potential toxicity risk. Loss of cadherin-bound β-catenin results in cadherin degradation and disrupts adherens junction formation, leading to cell detachment, disordered tissue architecture, and increased cancer metastasis (139–143). Moreover, cadherin knockouts lead to disrupted tissue function and decreased survival in mice (144–148). Finally, mutations of the genes that encode for cadherins result in numerous genetic conditions with diverse phenotypes (149). Therefore, it is crucial to develop drugs that selectively target the cytoplasmic β-catenin pool while sparing the cadherin-bound pool of β-catenin either through compounds that can distinguish between the destruction complex-bound cytoplasmic versus membrane cadherin-bound β-catenin or the recruitment of distinct E3 ligases (in the case of a targeted degradation approach) that have preferential activity between these two pools. Efforts to preferentially disrupt β-catenin-mediated transcription versus its interaction with cadherins are complicated by the binding of both E-cadherin and TCF/LEF to the same sites in β-catenin with high affinities (low nM to pM) (138, 143, 150, 151). Moreover, in most cell contexts, the cadherin-bound pool of β-catenin significantly exceeds the cytoplasmic Wnt signaling pool (152–154). Several classes of β-catenin-targeting drugs have been explored, including compounds designed to disrupt protein interactions or promote its targeted degradation.

### Competitive and allosteric inhibitors

Small molecules have been developed to competitively inhibit β-catenin’s binding interfaces, disrupt its protein-protein interactions, and reduce Wnt signaling output (155–162). Examples include PRI-724, which blocks β-catenin’s interaction with the CREB-binding protein (CBP) co-activator (155), and Tegavivint, which disrupts β-catenin’s interaction with the transcriptional co-activator TBL1 (156, 157). In addition to small molecules, peptides derived from β-catenin’s binding partners can act as competitive inhibitors (163). A notable example is SAH-BCL9, a peptide modeled after the BCL9 helix, which binds β-catenin’s armadillo repeat groove, selectively suppressing β-catenin–driven transcription and inhibiting Wnt-dependent tumor growth in preclinical models (164). While therapeutic peptides face challenges in delivery and stability, their high specificity offers advantages. Notably, many small-molecule inhibitors, including those competing with TCF, do not disrupt all β-catenin interactions, demonstrating the feasibility of selectively inhibiting TCF (162–164). However, achieving complete and selective inhibition remains challenging, as β-catenin interacts with multiple partners, meaning that blocking a single interface may only partially suppress Wnt signaling, limiting the efficacy and optimal therapeutic dosing of these compounds (**Table 1**).

**Table 1:** Summary of current β-catenin targeting drug classes.

Drug class	Name	Mechanism	Model systems tested	Clinical trials	β-Catenin/E-Cadherin Interaction	EC50 range	Citations
Competitive/Allosteric Inhibitors	FOG-001 ST316	Binds to β-catenin and prevent its interactions with other binding partners	CRC, Desmoid Tumor Monolayer Cells (CRC, Desmoid Tumor, Squamous Carcinoma, Gastric Cancer), Subcutaneous CRC Xenograft (Mice), *APC^min/+^* mice, Explant Patient Tissue Culture	Phase I/II trials, mixed efficacies and notable side effects	Does not disrupt	High nM-High µM	(136, 155–157, 159–164)
PROTACs	xStAx-VHLL C-Arg9-APCR3-VHL	Binds E3 ligase and protein of interest	CRC Monolayer cells, Patient-Derived CRC Organoids, Patient-Derived CRC Xenografts, CRC Subcutaneous Xenograft (Mice), *APC^min/+^* mice	Preclinical Studies	Not Tested	µM	(165–167)
β-catenin Destabilizing Proteins	C2 MSAB EN83	Allosterically binds β-catenin, resulting in β-catenin destabilization	CRC Monolayer Cells, Intestinal Organoids, CRC and Non-Small Cell Lung Cancer Subcutaneous Xenografts (Mice)	Preclinical Studies	Only one compound, MSAB, was tested and did not disrupt.	Low µM	(174–176)
Molecular Glues	NRX-252114	Induce or stabilize interaction between the protein of interest and the E3 ligase	HEK293T and Endometrial Adenocarcinoma Cells	Preclinical Studies	Not Tested	High µM	(177)
Ubiquibodies	Ecad-9-CHIPΔTPR Ecad-30-CHIPΔTPR	A fusion protein combining the E3 ligase with the binding domain for the protein of interest	CRC Monolayer Cells, WT Mice	Preclinical Studies	Does not disrupt	Not Tested (Expression Plasmids Used)	(179)
Condensate Modulators	DPTX3186	Sequester β-catenin in inactive condensates	Both *in vitro* and *in vivo* models	Phase I Clinical Trials	Not Tested	Not Reported	(180)
Antisense Oligonucleotides	PNA1	Bind to the mRNA of the protein of interest, promote mRNA degradation	CRC Monolayer Cells, CRC Subcutaneous Xenograft (Mice)	Preclinical Studies	Does not disrupt	µM	(181–183)

Mechanism of action, model systems tested, preclinical study results, effect on β-catenin-E-cadherin interaction, and EC50 range for β-catenin degradation *in vitro*. “Does not disrupt” indicates compound that does not disrupt β-catenin-E-cadherin binding.

### Targeted β-catenin degradation via PROTACs

Targeted degradation, including the proteolysis-targeting chimera (PROTAC) class of drugs, has emerged as a promising strategy to target non-enzymatic proteins such as β-catenin (165–168). PROTACs, the most extensively studied class of β-catenin inhibitors, recruit a target protein to an E2/E3 ubiquitin ligase complex to facilitate proteasomal degradation (169). PROTACs are effective against proteins lacking enzymatic grooves and primarily target cytosolic/nuclear proteins, potentially sparing membrane-associated β-catenin due to steric hindrance of the ternary drug complex at the protein-dense membrane (170, 171).

The first β-catenin PROTAC, xStAx-VHLL, linked a β-catenin-binding peptide (xStAx) derived from Axin to a VHL E3-ligand. This PROTAC achieved sustained β-catenin degradation, robust Wnt pathway inhibition, and suppressed growth of *APC*-mutant colorectal cancer (CRC) organoids and tumors in *APC^min/+^* mice (165). In 2024, nanoengineered peptide PROTACs (NP-PROTACs) were developed to simultaneously degrade β-catenin and STAT3, leveraging synergy between these oncogenic pathways that compensated for each other’s inhibition (166). NP-PROTACs were constructed by conjugating peptide binders for β-catenin and STAT3 to a lipid-PEG scaffold, forming nanoparticles to enhance cell delivery. These NP-PROTACs were shown to reduce the growth of CRC patient-derived organoids (PDOs) and patient-derived xenografts (PDXs). In 2025, Luo et al. (167) reported a novel bifunctional peptide PROTAC, C-Arg9-APCR3-VHL, targeting β-catenin in *APC*-mutant CRC. This chimera utilizes an APC-derived fragment to bind β-catenin, which is linked to a VHL-recruiting ligand featuring a poly-arginine tag to facilitate cell penetration. C-Arg9-APCR3-VHL induced β-catenin ubiquitination and proteasomal degradation, halting proliferation and inducing G1 arrest in *APC*-mutant CRC cells. In mice, it suppressed tumor growth, inhibited cancer migration, and reduced intestinal polyps in *APC^min/+^* models without observable systemic toxicity. Despite these advances, the current reported β-catenin PROTACs exhibit EC50s in the μM range (165–167). Therefore, there is a need to generate more potent β-catenin PROTACs that function at clinically relevant concentrations in the nanomolar or lower ranges. Moreover, their impact on membrane-bound, cadherin-associated β-catenin versus cytoplasmic β-catenin remains untested.

The dual roles of β-catenin have driven the development of compartment-selective interventions to limit degradation to the nucleus and cytosol, thereby preserving adherens junctions. One approach involves restricting β-catenin degradation to specific cellular compartments to avoid disrupting its membrane-associated pool. For instance, a CRBN or VHL E3-ligand could be modified with a nuclear localization signal, enabling PROTAC to target β-catenin for degradation exclusively in the nucleus. Another strategy leverages endogenous nuclear E3 ligases, such as TRIM33 or c-Cbl, which would bind and ubiquitinate nuclear β-catenin independently of its phosphorylation state (172, 173). These emerging approaches, still under development, underscore a shift toward location-specific protein degradation to enhance precision and minimize side effects for multifunctional proteins such as β-catenin.

### Other β-catenin targeting technologies

Other classes of protein degradation drugs that target β-catenin have also been explored. Small molecules that allosterically bind β-catenin can induce proteasomal degradation (174, 175). Similarly, small molecules that covalently bind β-catenin destabilize the protein, promoting its proteasomal degradation (176) without requiring an exogenous E3 ligand. Molecular glues represent another approach. These are monovalent drugs that induce or stabilize interactions between a target protein and an E3 ligase. A significant advance in this field is NRX-252114. This molecular glue, developed in 2019, binds the E3 adapter β-TrCP at its phosphoserine-binding site, enabling recognition of mutant β-catenin lacking the typical phosphodegron at its N terminus (177). While molecular glues offer improved pharmacokinetic properties compared to PROTACs due to their smaller sizes, their efficacy in β-catenin degradation and Wnt inhibition remains unexplored, particularly *in vivo*. Autophagy-mediated degradation of β-catenin is also under investigation, with reports indicating that β-catenin can be engulfed by autophagosomes through an LC3 interaction in specific contexts (178), suggesting potential for drugs that enhance this process. In addition, a β-catenin ubiquibody, a fusion protein combining an E3 ligase domain with a β-catenin-binding domain (e.g., an antibody fragment), has been developed. This antibody exhibits selectivity, degrading cytoplasmic and nuclear β-catenin while sparing membrane-associated β-catenin (179). Moreover, a β-catenin inhibitor developed by Dewpoint Therapeutics (DPTX3186), utilizes a novel mechanism of action: it sequesters β-catenin into inactive nuclear condensates (180). DPTX3186 is currently in Phase I clinical trials for Wnt-driven solid tumors including colorectal cancer, gastric cancer, lung cancer, and triple negative breast cancer (Clinical Trials.gov ID NCT07312903).

Finally, antisense oligonucleotides (ASOs) represent an additional approach to target β-catenin at the mRNA level. By binding to *CTNNB1* mRNA, ASOs trigger mRNA degradation and prevent translation, reducing β-catenin protein levels across cellular compartments (181, 182). As of 2025, no β-catenin-targeting ASO has entered clinical trials, but this approach could circumvent the need to identify ligandable pockets on β-catenin. Of note, one study used an antisense oligonucleotide that blocks splicing, generating a truncated β-catenin that retains α-catenin and E-cadherin binding but lacks its transactivating functions (183). Challenges with ASOs that continue to persist in their use as therapeutic agents include lack of stability, off-target effects, and poor cell uptake (184).

## Discussion

Therapeutic targeting of β-catenin has advanced significantly in recent years, transitioning from indirect modulators, upstream Wnt inhibitors, and broad general transcription blockers to precise, direct strategies. This review highlights the shift towards direct β-catenin elimination and compartment-selective pharmacology, moving beyond the upstream components in the Wnt pathway emphasized in earlier studies. Key innovations include novel PROTACs, molecular glues, and ubiquibodies that effectively degrade β-catenin in *APC*-mutant colorectal cancers, alongside peptide and small-molecule inhibitors advancing into clinical trials. Enhanced understanding of β-catenin’s distinct cellular pools has further refined these targeting strategies. Should membrane-sparing degradation prove viable, β-catenin could serve as a paradigm for subcellular precision in protein degraders, with transformative implications for targeted therapeutics across a range of diseases.
